# *Acanthus mollis* Formulations for Transdermal Delivery: From Hydrogels to Emulsions

**DOI:** 10.3390/gels10010036

**Published:** 2023-12-31

**Authors:** Patrícia Matos, Maria Teresa Batista, Francisco Veiga, Artur Figueirinha, Ana Figueiras

**Affiliations:** 1 University of Coimbra, Faculty of Pharmacy, 3000-548 Coimbra, Portugal; patricia_matos_20@hotmail.com (P.M.); fveiga@ci.uc.pt (F.V.); 2 University of Coimbra, LAQV, REQUIMTE, Faculty of Pharmacy, 3000-548 Coimbra, Portugal; 3 Chemical Process Engineering and Forest Products Research Centre (CIEPQPF), Department of Chemical Engineering, Faculty of Sciences and Technology, University of Coimbra, 3000-548 Coimbra, Portugal; mtpmb@ff.uc.pt

**Keywords:** *Acanthus mollis* L., DIBOA (2,4-dihydroxy-1,4-benzoxazin-3-one), topical formulations, hydrogels, emulsions

## Abstract

Topical formulations of *Acanthus mollis* L. leaf and the optimization of the release of their active compounds and their topical bioavailability were investigated for the first time. In vitro, the release of active compounds from three formulations—an oil-in-water cream and two hydrogels (Carbopol 940 and Pluronic F-127)—was determined using Franz diffusion cells. Detection and quantification of the compounds was performed via high-performance liquid chromatography with a photodiode array (HPLC-PDA). DIBOA, a bioactive compound of this medicinal plant, exhibited release kinetics of the Weibull model for the Carbopol and Pluronic F-127 formulation, identifying it as a potential active agent to optimize the topical distribution of the formulations. The implications extend to applications in inflammation treatment and tyrosinase inhibition, suggesting that it can make a significant contribution to addressing skin conditions, including melanoma and various inflammatory diseases.

## 1. Introduction

*Acanthus mollis* L. is a member of the Acanthaceae family and is native to the Mediterranean region. Traditionally, *Acanthus mollis* leaf has been used to treat wounds, burns, sprains, fractures, and bruises [1,2], as well as to alleviate swollen legs and headaches [3]—conditions involving inflammatory processes. Gargles with plant extracts also relieve toothache and mouth inflammation [2,4]. Over the last few years, some studies have confirmed the anti-inflammatory activity of *A. mollis* leaf extracts. A methanol extract increased the biosynthesis of 15 (S)-HETE, an anti-inflammatory eicosanoid [5], whereas an ethanolic extract at a concentration of 15 μg/mL inhibited nitric oxide (NO) production safely in the mouse macrophage (RAW 264.7) cell line [6,7]. Previous studies have provided compelling evidence of the anti-tyrosinase activity exhibited by the ethanolic extract of *A. mollis* leaf and its phytoconstituents [8]. This finding indicates that the bioactive compounds present in the plant, when extracted and studied individually and collectively in the form of the ethanolic extract, contribute to inhibiting the activity of the enzyme tyrosinase, which is often elevated in melanoma cells [9]. This increased activity can contribute to the uncontrolled synthesis of melanin and may also play a role in the resistance of melanoma cells to specific treatments. Exploring targeted therapies to disrupt the melanin synthesis process and inhibit melanoma growth is crucial. Research into tyrosinase inhibitors has gained significance in the context of skin cancer prevention and treatment [10].

Although the plant is commonly used in folk medicine for direct application through poultices to facilitate wound healing and alleviate inflammation, the extracts can pose challenges to this topical administration. Additionally, there may be other adverse effects or injuries associated with this application. Topical administration can be an alternative to different routes, as it avoids the first-pass effect and is generally well tolerated [11]. In this sense, to improve its characteristics, enhance its pharmacological effects, and reduce its adverse effects, pharmaceutical forms have been developed. Semi-solid preparations, such as ointments, creams, or gels, mainly represent formulations for topical application with local or systemic effects [12]. In addition to lotions and balms, which have disadvantages such as penetration into the stratum corneum, gelled systems stand out, as they are easy to prepare and apply, allowing prolonged active ingredient delivery [13].

**Figure 1:**
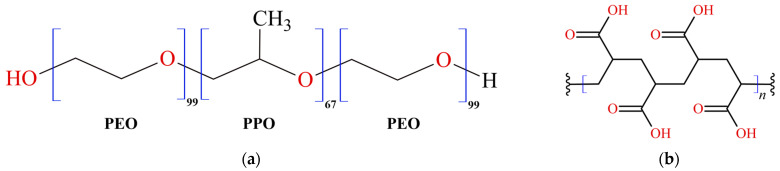
Chemical structures of polymers: (**a**) Pluronic F-127 and (**b**) Carbopol 940.

Within the pharmaceutical and cosmetic sectors, gelled systems are extensively utilized owing to their adaptability and the ability to customize release profiles. These systems are commonly employed in dermal and transdermal drug delivery, skincare formulations, and various cosmetic applications. Gelled systems, distinguished by their gel-like texture, can be personalized by choosing specific gelling agents, polymers, and additives to attain the desired viscosity, texture, and release attributes. Formulated by integrating a gelling agent or polymer into a liquid base, they yield semi-solid products with distinctive properties. These systems are known for their ability to maintain their structure without flowing, offering advantages in various applications. Hydrogels are polymeric systems that respond to external triggers such as temperature and pH [14]. Pluronic F-127 (PF127), or Poloxamer 407 (Figure 1a), is an amphiphilic block copolymer composed of ethylene (PEO) and propylene (PPO) oxide units. It is renowned for its ability to form temperature-sensitive gels. PF127 remains liquid at lower temperatures but transitions into a gel as the temperature increases. This property makes it suitable for in situ gel formation, where a liquid formulation turns into a gel upon application to the body [15]. Carbopol 940 (C940), known as polyacrylic acid (Figure 1b), is a synthetic polymer frequently used to create pH-sensitive gels. It is available as a dry powder that readily swells and forms a gel when dispersed in water or other aqueous solvents. The viscosity and consistency of the resulting gel depend on the concentration of Carbopol and the pH of the surrounding environment [16].

This study employs a systematic approach, involving variations in types and concentrations of polymeric carriers, to uncover how these factors influence the release characteristics of the phytochemicals in *Acanthus mollis* ethanolic extract (EEt). By doing so, the research aims to clarify how the chosen carriers and their proportions can alter the release kinetics of bioactive compounds from EEt, with potential implications for its therapeutic effectiveness and suitability for use. The investigation delves into the in vitro release of DIBOA and verbascoside of EEt from diverse gel bases and creams for the first time. This exploration yields valuable insights that contribute to optimizing topical delivery systems. The knowledge gained from this research can play a pivotal role in formulating pharmaceutical products that ensure the controlled and sustained release of the active compound, thus elevating its therapeutic potential. In summary, this research centers on the intricate interplay between diverse polymeric carriers and varying concentrations to elucidate their impact on the release behavior of the phytoconstituents in EEt. The overarching goal is to enhance our understanding of how these factors influence the therapeutic efficacy and application suitability of EEt. Ultimately, the study offers valuable insights for refining topical delivery systems and formulating effective pharmaceutical products.

## 2. Results and Discussion

### 2.1. Pre-Screen Solubility Studies

The development of a formulation necessitates the prior selection of pharmaceutically acceptable, non-irritating and non-sensitizing excipients. These excipients should be deemed safe (GRAS status) and suitable for the chosen delivery route [17]. Screening components for preparing formulations requires a gradual selection of liquids based on the relative solubility of the extract—an essential factor for encapsulation efficiency. High solubility is anticipated to result in high encapsulation efficiency.

**Figure 2:**
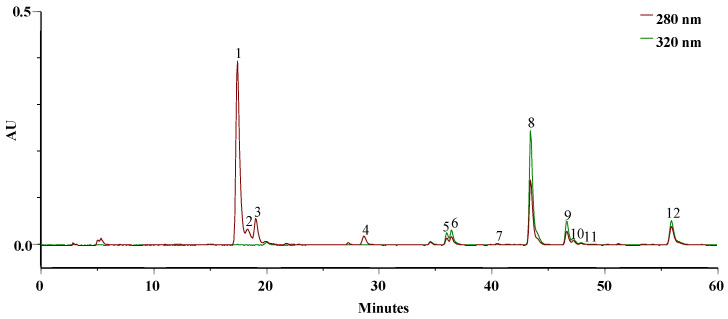
HPLC–PDA profile of the EEt (3.14 mg/mL) from *A. mollis* leaf at 0 min in PBS, recorded at 280 and 320 nm.

Figure 2 displays the typical chromatogram of the ethanolic extract solubilized in PBS at pH = 7.4 at 0 min, resembling the simulation of blood in the systemic circulation of the active principles. 

**Figure 3:**
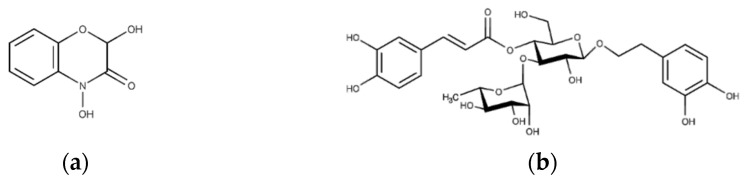
Structure of the compounds studied: (**a**) 2,4-dihydroxy-1,4-benzoxazin-3-one (DIBOA) and (**b**) verbascoside.

The extract demonstrated solubility in PBS, and the primary bioactive phytoconstituents of this extract [6], namely, DIBOA (Figure 3a) and verbascoside (Figure 3b), represented by peaks **1** and **8**, respectively, were detected.

In addition to these, we identified a derivative of DIBOA (**2**), HBOA (**3**), and BOA (**4**), as well as β-OH-verbascoside (**5**, **6**), and two isomers of β-EtOH-verbascoside (**9**, **10**). Furthermore, an isomer verbascoside (**11**) and hispidulin glucuronide (**12**) were present. Compounds **1**, **3**, and **8** were confirmed with authentic standards, whereas the remainder were identified by mass spectroscopy only.

**Figure 4:**
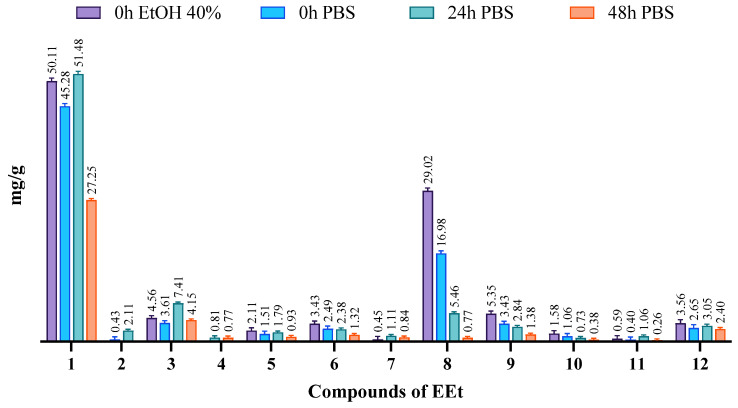
Evaluation of the solubility of the phytoconstituents of EEt in 40% EtOH and PBS medium at 37 °C at different time points.

The ethanolic extract, comprising a mixture of several compounds, exhibited poor solubility in water. To assess solubility, we conducted phase solubility studies in water, 40% ethanol, and PBS pH = 7.4, all at 37 °C. The solubilities of the phases were determined by shaking bottles containing an excess of extract and the various solvents for 48 h, following the guidelines of the European Pharmacopeia [18]. Subsequently, HPLC-PDA analyzed and quantified the phytoconstituents in these mixtures using calibration lines of the compounds in the extract. In Figure 4, chromatograms depict the extract solubilized in 40% ethanol and PBS medium at 37 °C. It is evident that DIBOA (compound **1**) was highly soluble in ethanol and PBS, with complete solubility in PBS achieved after 24 h. However, for verbascoside (compound **8**), solubility in PBS was relatively low, and even with time there was minimal increase. Notably, after 48 h, all compounds exhibited a significant decrease in solubility, which may be attributed to the degradation of these compounds.

Although verbascoside did not demonstrate excellent solubility in the solvent used for the formulations, we proceeded similarly, as DIBOA was previously identified as the compound that exhibited significant anti-inflammatory activity at a concentration devoid of cytotoxicity. Moreover, DIBOA has shown activity on tyrosinase [8], an essential precursor of melanin that is overexpressed in melanogenesis.

Verbascoside had a solubility of approximately 10 mg/mL in PBS (pH 7.4), which was less than in ethanol solvent, where it reached 30 mg/mL.

### 2.2. ATR-FTIR Measurements

**Figure 5:**
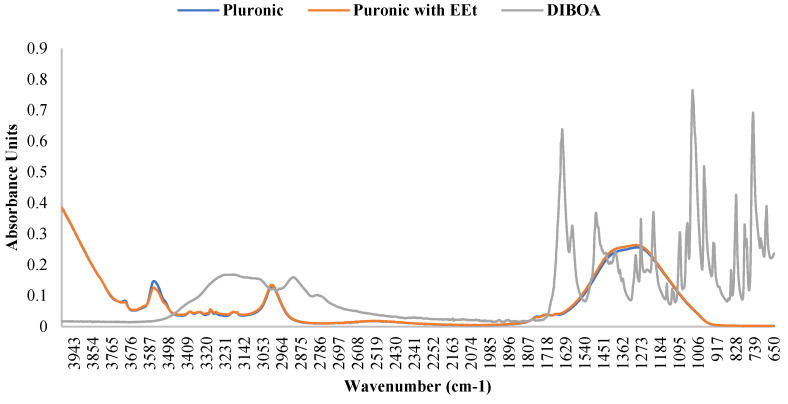
FTIR spectra of Pluronic F-127, Pluronic F-127 with EEt, and DIBOA.

According to the authors of [19,20,21], the presence of water affects the infrared (IR) bands in PF127 spectra, revealing changes in molecular vibrations influenced by hydration. Generally, water weakens and broadens the bands, sometimes altering their frequency and merging them into a broader band. The C-O-C stretching bands change, blending into a single broadband with a frequency shift from 1106 to 1084 cm^−1^. This is mainly attributed to the formation of hydrogen bonds between the oxygen atoms in the ether structure of PF127 and water molecules. CH_2_ torsional vibrations shift to higher frequencies in the presence of water, while the C-O-C stretching bands remain unchanged. The oscillating vibration of CH_2_ also increases in frequency. The symmetric strain band of CH_3_ widens and weakens in the presence of water. In an aqueous solution, a combination of two bands can be considered, one representing hydrated methyl groups and the other methyl groups in the anhydrous state. Differences in the spectra are most evident in the dry form, and water dominates the spectra in the range of 1500–1800 cm^−1^. However, incorporating the extract into the 22% PF127 formulation did not alter the infrared spectrum of pure PF127, as seen in the figure. The bands associated with the main functional groups of Pluronic confirmed the encapsulation of the EEt into micelles without changes in chemical bonds. The attenuation in the intensity of the peaks at 3512 cm^−1^ suggests the formation of intermolecular hydrogen bonds between the EEt –OH groups and POE groups in the Pluronic corona. This indicates that the presence of the extract did not cause significant changes in the IR bands of PF127 under the conditions tested. This may be important to ensuring the stability and integrity of the properties of PF127 when used as a vehicle for the extract. Figure 5 shows the infrared spectrum of DIBOA. Absorbance at 3289 cm^−1^ (br, OH), 3150 cm^−1^ (br, Ar-H), 2940 cm^−1^ (sh), 1663 cm^−1^ (CO), and 1603 cm^−1^ (aromatic) are characteristic of this type of compound [22].

Pluronic’s ATR-FTIR spectra with EEt were compared with those of blank Pluronic, as shown in Figure 5. The spectra of the EEt formulation showed the absence of peaks beyond those characteristic of the free Pluronic formulation, suggesting that the EEt was localized and trapped in the hydrophobic micellar core.

### 2.3. Texture Analysis of Carbopol Formulations

**Figure 6:**
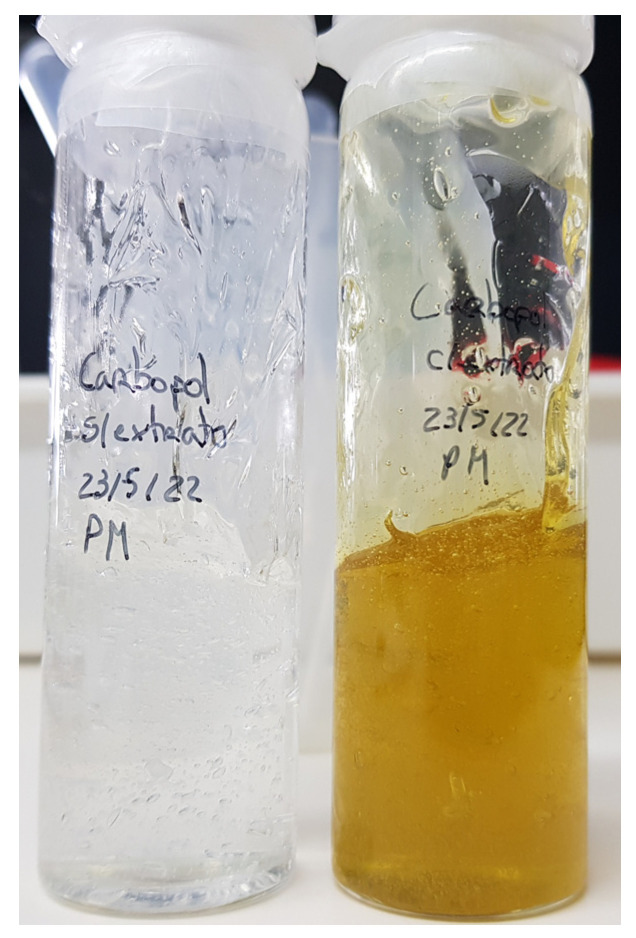
The physical appearance of hydrogels without and with EEt.

The assessment of a cosmetic product’s texture is crucial for its characterization and user acceptance, influencing ease of handling. When hydrogels are applied to the dermis, they need to establish a microgel network capable of withstanding physiological stress caused by body movement while maintaining close and sustained contact with the skin. Figure 6 illustrates the visual characteristics of the Carbopol formulation both without and with smaller quantities of the extract.

Maintaining a balance between gel cohesion and adhesiveness is crucial for creating an ideal topical formulation, especially for hydrogels intended for prolonged retention at the application site in topical treatments. Texture analysis provides a reliable summary of these qualities. Cohesiveness, adhesiveness, and hardness are analyzed through penetration tests, wherein a probe (with appropriate dimensions and characteristics) penetrates the sample at a pre-determined speed and distance. Subsequently, it returns to the initial position to measure the resistance offered by the material in question [23].

**Figure 7:**
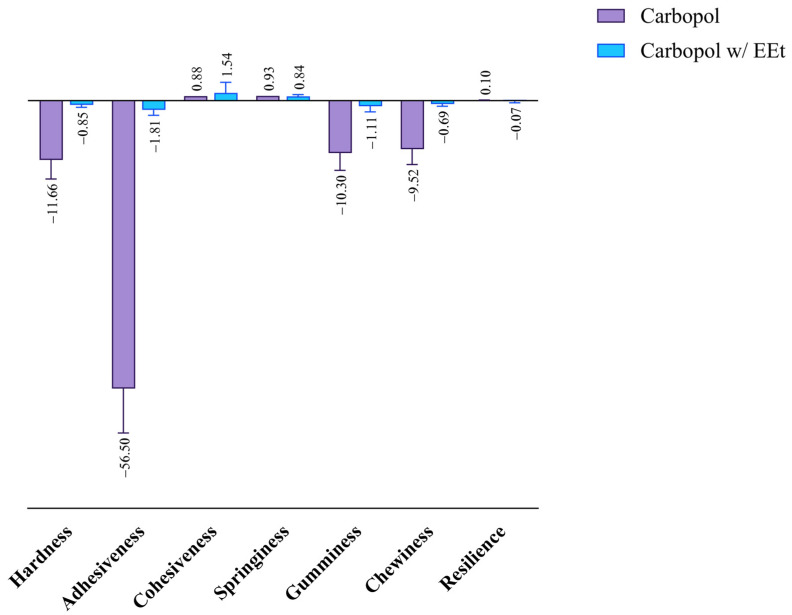
Effect of ethanolic extract in Carbopol 940 on the formulations’ mechanical properties.

Cohesiveness refers to the area of positive force during the first and second compression. It can be measured as the rate at which the material disintegrates under mechanical action, with tensile strength manifesting this parameter. The results obtained after studying the cohesiveness of Carbopol formulations with EEt are shown in Figure 7. The figure indicates that the formulations exhibited similar cohesiveness to the formulation without extract, demonstrating superior resistance to disintegration under mechanical action.

Adhesiveness is linked to the adhesive properties demonstrated by gels, thereby influencing the length of time the formulation remains at the application site. It can be defined as the area of opposing force between compressions, representing the work necessary to overcome the traction forces between a product’s and piston’s surfaces. Adhesiveness consists of the total force required to pull the compression piston from the sample. From the analysis of the figure, it is evident that formulations with extract exhibited less adhesiveness than Carbopol alone, indicating a shorter residence time at the application site with higher extract concentrations. Adhesiveness is closely related to bioadhesion, and a higher value is desired to increase the retention time on the skin.

Hardness refers to the maximum peak force during the first compression cycle (first bite) and is often interchangeably used with firmness. By analyzing the figure, a similar behavior to the previous parameter can be observed. Formulations with extract had a lower hardness than Carbopol alone. A lower hardness value indicates better applicability to the skin, facilitating easy administration and spreadability.

Incorporating an extract into a Carbopol-based formulation has profound implications for mechanical properties, requiring a nuanced consideration of various factors. The hydration level, a primary influencer, plays a pivotal role in shaping the mechanical characteristics of Carbopol hydrogels. Due to their sensitivity to water content, these hydrogels undergo changes in stiffness and elasticity with fluctuations in hydration. Introducing an extract can modulate the hydration level, impacting overall mechanical properties and potentially leading to decreased associated values. Crucial considerations extend to the interactions between the extract and the Carbopol polymer network, influencing both structure and mechanical properties. Additionally, the frequent use of Carbopol to enhance formulation viscosity becomes intricately connected with mechanical behavior. Alterations induced by the extract can affect mechanical strength, emphasizing the interconnected nature of viscosity and mechanical properties. The concentration of the extract and Carbopol emerges as a critical determinant of mechanical properties, making it imperative to strike the right balance to achieve the desired characteristics. In summary, a comprehensive understanding and deliberate manipulation of these factors are essential for optimizing the mechanical performance of Carbopol-based formulations with incorporated extracts.

### 2.4. In Vitro Drug Release Studies

**Figure 8:**
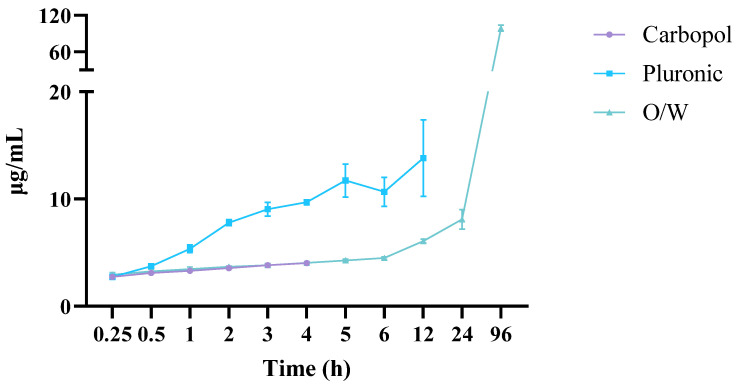
The release profiles of the Carbopol, Pluronic, and oil–water cream with the ethanolic extract considering its main compound, DIBOA.

The Franz vertical diffusion cell monitored the diffusion of C940, PF127, and oil–water (O/W) cream formulations at 37 °C. HPLC-PDA quantified the amount of EEt that diffused from the hydrogel in the donor chamber through the membrane to the recipient chamber using the calibration lines (Figure 8).

The PF127 formulation, with the lowest concentration of just 10%, exhibited free-flowing characteristics similar to water at the formulation temperature and could even adapt to the testing temperature. This suggests that this formulation may not exhibit gel characteristics at test temperatures. However, the formulation with a higher concentration of PF127, namely, 22%, was able to flow freely at 5 ± 1 °C but transformed into a rigid gel at 27 ± 1 and 37 ± 1 °C. This observation aligns with previous work reported by Vadnere and collaborators (1984) [24]. The phenomenon can be explained by changes in the properties of the poly(ethylene oxide) and poly(propylene oxide) (PEO and PPO, respectively) domains within PF127 molecules following alterations in concentration or temperature in the Pluronic solution system [25]. Below the critical concentration of micelles, PEO and PPO in the Pluronic molecules were hydrated. As the temperature increased, the PPO chains became less soluble than PEO and dehydrated. This led to hydrophobic interactions between the PPO domains, resulting in the formation of spherical micelles with a dehydrated PPO core surrounded by an outer layer of hydrated and swollen PEO. Consequently, the micelles hardly occupied a high solution fraction, limiting contact and entanglement and resulting in a three-dimensional network structure and the formation of a rigid gel [25,26]. Therefore, products with a higher effective concentration of PF127 would contain more micelles, requiring less energy to promote the sol–gel transition and enabling the sol–gel change at lower temperatures than products with lower PF127 content.

The examination of drug release profiles across various formulations involved the utilization of mathematical models, with the computation of each model’s correlation coefficient (r^2^), presented in Table 1. For the Carbopol formulation, these models were applied for up to 4 h. Subsequent observations indicated a decrease in the released DIBOA concentration, suggesting potential degradation of the free drug within the hydrogel upon contact with the dissolution medium.

The zero-order model proposes a consistent release rate over time, independent of the remaining drug quantity in the formulation. This model is suitable for scenarios where factors beyond drug concentration predominantly influence the release rate [27]. It finds relevance in systems governed by factors like drug delivery system design, formulation properties, or specific release control mechanisms. This relationship is instrumental in describing modified and prolonged drug release, as seen in transdermal systems [28]. In contrast, the first-order model assumes a drug release rate directly proportional to the remaining amount of drug in the formulation, leading to a diminishing release rate over time as the drug concentration decreases. This model implies an inherent dependency on the drug concentration in the formulation and is valuable for understanding drug release mechanisms influenced by factors such as dissolution, diffusion, or other release control mechanisms [29]. The Higuchi model is applicable for evaluating the release of poorly soluble drugs in solid, inert, and insoluble matrices, particularly when drug release is mainly governed by a diffusion process. Based on Fick’s law of diffusion, the model assumes that the drug release rate is directly proportional to the square root of time [30]. This suggests a slower and more controlled release of the drug over an extended period. The Hixson–Crowell model is commonly used when there is a change in the surface area and diameter of drug particles over time, especially in scenarios where the drug release mechanism is primarily governed by particle size reduction or dissolution. It assumes that the drug release is proportional to the cube root of the remaining drug volume, indicating a decrease in the release rate over time [31]. The Korsmeyer–Peppas model is widely used to describe drug release from polymeric systems and other complex formulations. Derived from the power law relationship, this empirical equation is frequently employed to analyze release mechanisms where Fickian diffusion is not the sole governing factor. The model’s versatility makes it valuable in developing and optimizing drug delivery systems [32]. The Weibull model is flexible and adaptable to fit a wide range of release profiles, making it valuable in situations where other models may not be as applicable. It is advantageous for dealing with complex release mechanisms exhibiting non-linear and non-Fickian behavior [33]. The Baker–Lonsdale model, developed from the Higuchi model, describes drug release from spherical matrices and is particularly useful for linearizing release data from various formulations of microcapsules or microspheres [34].

Upon scrutinizing the calculated correlation coefficient values, it becomes evident that the Carbopol and Pluronic formulations exhibited a release profile closely aligned with kinetics resembling the Weibull model. The Weibull model showcases characteristic behavior in drug release kinetics, offering a versatile framework to describe the variability in the time it takes for a drug to be released from a formulation. Notably, its flexibility allows it to adapt to different shapes in its probability density function, effectively capturing diverse release profiles. The oil/water cream release profile demonstrated coefficient values that closely approximate the zero-order and Hixson–Crowell models. The zero-order model’s distinctive feature is its ability to describe a constant release rate throughout the release duration, providing a predictable and controlled manner of delivering the drug. This characteristic behavior is valuable for designing drug delivery systems that aim to avoid fluctuations in plasma concentrations, reduce side effects, and enhance patient adherence by minimizing the frequency of administration. The Hixson–Crowell model proposes that the drug dissolution rate is directly proportional to the cube root of the remaining drug mass, implying that the drug release process is governed by changes in the drug’s surface area as it dissolves. In practical terms, this model is well suited for formulations where the primary drug release mechanism involves the dissolution of solid particles. Despite their distinct formulations, the zero-order and Hixson–Crowell models share similarities in their applications and implications within drug release kinetics. Both models describe scenarios where the rate of drug release remains constant over time, contributing to their everyday use in the context of sustained-release formulations. The zero-order model, characterized by a continuous release rate independent of remaining drug concentration, finds practical application in sustained drug delivery systems, achieving a consistent and predictable release profile. Similarly, the Hixson–Crowell model, specifically designed for solid dosage forms, is also applied in sustained-release scenarios where changes in surface area during dissolution influence drug release kinetics. Researchers navigate between these models based on the specific characteristics of the drug delivery system under investigation, choosing the one that best aligns with the observed mechanisms and goals for sustained drug release. Incorporating poloxamers in hydrogel preparation facilitates the creation of semi-solid formulations for the controlled release of the active ingredient. This, in turn, enables the extension of the therapeutic effect, mitigating peaks in plasma concentrations and related adverse effects while concurrently diminishing the dosage and frequency of drug administration. These factors significantly contribute to an elevation in drug bioavailability, correlating with an enhancement in therapeutic adherence.

### 2.5. Microscopic Analysis

**Figure 9:**
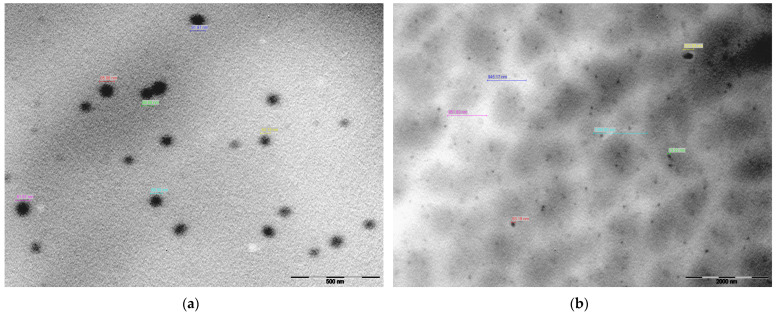
Characterizing the Pluronic F-127 hydrogel by transmission electron microscopy (TEM): (**a**) hydrogel without EEt; (**b**) hydrogel loaded with EEt.

Figure 9 presents TEM morphological images of PF127 in the presence and absence of EEt. Pluronic^®^ F-127 spontaneously formed spherical micelles in an aqueous solution at its critical micellar concentration (CMC) [35,36]. TEM analysis of the hydrogel loaded with EEt revealed slightly larger micelles compared to those without the extract (900–1300 nm and 54–85 nm, respectively). These larger micelles exhibited a more pronounced negative contrast on the periphery of the spheres, likely due to the distribution of the EEt (Figure 9b). In contrast, the PF127 hydrogel without EEt (Figure 9a) displayed uniform micellar structures similar to the control. For a deeper understanding of size and morphological variations, it is advisable to perform thermal analyses of the hydrogel using TGA (thermogravimetric analysis) and DSC (differential scanning calorimetry). TGA and DSC are instrumental techniques in studying and characterizing hydrogels, providing valuable information about their thermal properties, composition, and behavior. TGA investigates a hydrogel’s weight changes as a function of temperature. This technique is crucial for assessing the thermal stability of hydrogels and determining the onset of decomposition or degradation. By analyzing weight loss profiles, TGA helps identify the temperature range within which significant changes occur, aiding researchers and formulators in establishing the optimal conditions for storing, handling, and applying hydrogel-based products. Complementing TGA, DSC is utilized to study the heat flow associated with phase transitions and chemical reactions within hydrogels. This includes the investigation of gelation and melting processes, providing insights into the material’s structural changes and thermal transitions. The combined application of TGA and DSC offers a holistic view of hydrogels’ thermal behavior and composition. These techniques are crucial for assessing the presence of water or other volatile components within the hydrogel matrix, observed through weight loss in TGA or shifts in heat flow in DSC. Understanding these aspects is essential for maintaining the integrity and functionality of hydrogel-based products. Moreover, TGA and DSC contribute significantly to hydrogel manufacturing’s quality control and batch-to-batch consistency. Monitoring thermal properties ensures that hydrogel formulations meet predefined specifications, providing confidence in the reproducibility and reliability of the final products.

## 3. Conclusions

This study marks the initial exploration of a topical formulation incorporating a bioactive *A. mollis* leaf extract. Two natural active compounds from the plant, DIBOA and verbascoside, were identified as pertinent elements for skin permeation studies. The absorption and penetration of these compounds into the skin, together with their established biological activities, position them as promising candidates for developing new and potent anti-inflammatory, antioxidant, and anti-cancer formulations. In this study, it was concluded that the Pluronic formulation emerged as the optimal choice for releasing DIBOA. This formulation revealed a kinetic Weibull model. Overall, the study presents a practical approach to designing a range of topical medicines that preserve the valuable herbal ingredients appreciated by many patients while achieving high standardization and quality compared to existing market offerings.

## 4. Materials and Methods

### 4.1. Materials

Carbopol-940^®^ (Carbomer 940) and Pluronic F-127^®^ (Poloxamer 407) were obtained from Fagron Iberica (Barcelona, Spain). Stearic acid was provided by VWR Chemicals (Leuven, Belgium), and triethanolamine was purchased from Panreac AppliChem (Darmstadt, Germany). Glycerin was obtained from VWR Chemicals (Leuven, Belgium), and methylparaben was sourced from Merck (Burlington, MA, USA). Water was purified (Millipore^®^) and filtered through a 0.22 mm nylon filter before use. All solvents used were HPLC grade, whereas all other materials were of analytical grade.

### 4.2. Ethanol Extract

The leaves of *A. mollis* were collected from Coimbra city (40°12′00″ N, 8°25′02″ W) in February 2018, and a voucher specimen (A. Figueirinha 01015) has been archived at the Herbarium of Medicinal Plants, Faculty of Pharmacy, University of Coimbra. To preserve the plant material’s integrity, it was refrigerated at −20 °C and shielded from light and moisture until needed. Extraction of the ethanolic extract (EEt) from the leaves involved stirring 4 g of plant material with 96% ethanol (200 mL) for one hour using an electromagnetic stirrer. After filtration, water was introduced, and the extract was left overnight in a cold environment. Subsequently, the mixture was centrifuged to eliminate chlorophyll, and the resulting supernatant was collected for further use. The composition had been previously identified by Matos and collaborators [8].

### 4.3. Solubility Studies

The solubility of the extract in water and PBS was initially determined as a prerequisite for formulation. For PBS solubility at pH = 7.4, the extract was converted to dry residue and dissolved in 2.5 mL of PBS. Samples were dispersed in screw-cap tubes with solvents (5 mL each) and magnetically stirred for 48 h at 25 °C. Collection points were set at 24 and 48 h, followed by filtration through a 0.22 µm membrane. Analysis and quantification were conducted using the HPLC-PDA Gilson system (Gilson Inc., Middleton, WI, USA), consisting of two pumps (models 305 and 306), a mixer (Model 811 B), a manometric module (model 805), and an autosampler (Gilson 234 autoinjector) coupled to a PDA (Gilson model 170). Data acquisition and control were managed through the Unipoint System data (Unipoint^®^ 2.10) at the control station. The analyses were performed on a Waters^®^ RP18 Spherisorb ODS-2 column (250 × 4.6 mm; 5 μm particle size), maintained at 35 °C, and protected by a KS 30/4 Nucleosil 120–5 C-18 Macherey-Nagel guard column (Duren, Germany). The mobile phase comprised a 5% aqueous formic acid solution (A) and acetonitrile (B), utilized at a flow rate of 1 mL/min. The gradient was programmed from 0% to 25% B over 0–62 min and 25% to 100% B at 65 min, followed by an isocratic phase for 10 min. UV-V profiles were acquired in the 200–600 nm range, and chromatograms were recorded at 280 and 320 nm. DIBOA and other compounds were quantified using an HPLC method as previously described [8]. Each determination was performed in triplicate.

### 4.4. Preparation of Ethanolic Extract Topical Gels and Emulsions

After conducting several preliminary studies, two hydrogel formulations were developed with the compositions outlined in Table 2. The hydrogels were prepared by dissolving the extract in water. For the C940 hydrogel, EEt gels were formulated with a concentration considering the maximum solubility of the primary compound, DIBOA, using C940 at a concentration of 0.5% (*w*/*w*). C940 powder was gradually added to distilled water and stirred using a magnetic stirrer (Jenway 1000, Jenway, Staffordshire, UK) until complete homogeneity was reached. Triethanolamine drops were added to adjust the pH of the gel to a range of 6.5 to 8.3. The pH was measured using a pH meter (BioBase pH meter model PHS-3BW, Jinan City, China) to ensure safety against irritation. The ethanolic extract was dissolved in the remaining distilled water and added to the gel.

For the PF127 hydrogel, EEt was formulated with PF127 at a concentration of 22% (*w*/*v*). PF127 was added to cold water (T = 5 °C, using an ice bath) and stirred with a magnetic stirrer until a precise, homogeneous liquid was formed. The weighted amount of extract was dissolved in the remaining distilled water and added to the liquid, which was stirred under cooling conditions for 12 h until a translucent and homogeneous solution was obtained. After this time, the formulation was left at room temperature to ensure a uniform gel formulation. If the gel did not form, the formulation was heated to 32 °C.

A cream based on an oil-in-water (O/W) emulsion (semi-solid formulation) was also prepared. The emulsifier (stearic acid) and triethanolamine were dissolved in the oil phase and heated to 70 °C. The preservative (methylparaben) and aqueous components (glycerin and water) were dissolved at 70 °C. The aqueous phase was added in aliquots to the oil phase with continuous stirring. The extract redissolved in a small amount of water was added after the temperature decreased. The details are provided in Table 1.

### 4.5. Texture Profile Analysis (TPA)

The textural characteristics of Carbopol, including hardness, compressibility, adhesiveness, cohesiveness, and elasticity, were assessed using the Texture Analyzer TA.XT Plus from Stable Micro Systems Ltd. (Surrey, UK). The TPA mode involved the use of an analytical probe (P/10, 10 mm Delrin). The probe underwent two compressions into the sample at a rate of 5 mm/s to a depth of 15 mm, with a 15 s delay between consecutive compressions. Six replicates were performed for each formulation at room temperature.

Data were collected and calculated using the Texture Exponent 3.0.5.0 software associated with the instrument. The force–time curve obtained facilitated the determination of various mechanical parameters, such as hardness (the maximum peak force during the first compression cycle), compressibility (the work required to deform the sample during the first compression, calculated from area under the curve 1 (AUC1)), adhesiveness (the work needed to overcome attractive forces between the sample and the probe surface, calculated from AUC2), cohesiveness (the ratio of the area under the force–time curve on the second compression cycle to that on the first compression cycle), and elasticity (the ratio of the time required for maximum structural deformation on the second compression cycle to that on the first compression cycle).

### 4.6. Physicochemical Evaluation of Prepared EEt Gels

#### 4.6.1. Attenuated Total Reflectance Fourier Transform Infrared

Attenuated total reflectance Fourier transform infrared (ATR-FTIR) spectra of gelled formulations were obtained using a Spectrum 400 instrument from Perkin-Elmer, Waltham, MA, USA. The ATR accessory was fitted with a Zn-Se crystal plate. Samples were placed in the ATR device, and measurements were conducted with 32 scans for each spectrum at a scan speed of 0.5 cm/s and a resolution of 2 cm^−1^. The spectra were collected in the wavenumber range of between 4000 and 650 cm^−1^.

#### 4.6.2. Transmission Electron Microscopy (TEM)

TEM was employed for morphological analyses of the Pluronic formulations. In brief, the mesh grid was immersed in alcian blue at 1% (*w*/*v*) for 10 min, thoroughly washed, and then stained in contact with the mesh grid for 1 min. A drop of the pre-treated sample was placed on a mesh grid and dried before visualization. Freshly prepared nanosystem solutions were placed on a formvar and carbon-coated copper grid, and the grid was dried for five min. Samples were analyzed at 120 kV, and images were captured with a digital camera system (MegaView III–SIS). The observations were carried out using a Tecnai G2 Spirit BioTWIN transmission electron microscope (FEI Company, Eindhoven, The Netherlands) at 100 kV.

#### 4.6.3. In Vitro Release Studies

**Figure 10:**
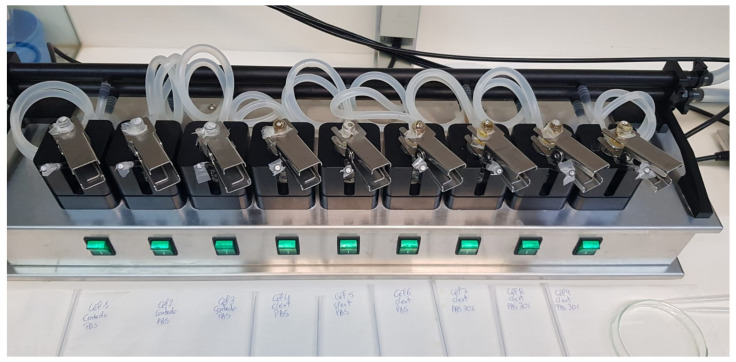
Multi-station Franz diffusion cell system.

In vitro release studies were conducted using static vertical Franz diffusion cells (PermeGear, Inc., Hellertown, PA, USA) equipped with a diffusion area of 0.636 cm^2^ and a 5 mL receptor compartment (Figure 10). Cellulose membranes with a molecular weight cutoff (MWCO) of approximately 12,000 and a mean flat width of 33 mm (D9652, Sigma-Aldrich, St. Louis, MO, USA) were utilized as artificial membranes. Additionally, Strat-M™ membranes from Merck Millipore (Burlington, MA, USA), designed to simulate the human skin, were employed. Each membrane was positioned between the two compartments, with a receptor solution consisting of phosphate buffer with pH 7.4, which was used to maintain sinking conditions. The receptor compartment was stirred at 600 rpm and maintained at a temperature of 32 ± 0.5 °C with a thermostatic water pump circulating water through each chamber jacket to mimic skin conditions. The formulations (1 g) were applied to the donor compartment. Subsequently, 1 mL of the receptor medium was collected at specific time intervals (15, 30, 60, 120, 180, 240, 300, 360, 720, and 1440 min), and each collected sample was immediately replaced by an equal volume of fresh solution. The collected samples were then analyzed for DIBOA content using the HPLC method.

The study employed various mathematical models to analyze the obtained results and predict the drug release mechanism from specific formulations. The investigation focused on understanding how DIBOA is released over time and under different conditions, which is a crucial aspect of pharmaceutical research and development. The models used in the investigation include the zero-order release model (1), first-order release model (2), Higuchi model (3), Hixson–Crowell model (4), Korsmeyer–Peppas model (5), Weibull model (6), and Baker–Lonsdale model (7). Each model represents different aspects of drug release kinetics, accounting for factors such as time, diffusion, erosion, and swelling in the formulation [27]. The models provide valuable insights into the release mechanisms of the formulations under investigation.

Mathematically, the models are represented as:

(1) $$Q\left(t\right)=Q_{0}-kt$$

(2) $$Q\left(t\right)=Q_{0}e^{-kt}$$

(3) $$Q\left(t\right)=kt^{0.5}$$

(4) $$Q\left(t\right)=Q_{0}{\left(1-\frac{t}{T}\right)}^{\frac{3}{2}}$$

(5) $$Q\left(t\right)=K\times t^{n}$$

(6) $$Q\left(t\right)=M\times \left(1-e^{-{\left(\frac{t}{\lambda }\right)}^{k}}\right)$$

(7) $$\frac{3}{2}\left[1-{\left(1-\frac{M_{t}}{M_{\infty }}\right)}^{\frac{2}{3}}\right]-\frac{M_{t}}{M_{\infty }}=k_{2}t$$

where

- Q(t) is the amount of drug released at time t;
- $Q_{0}$ is the initial amount of drug in the formulation;
- k is the zero-order/first-order/Higuchi release rate constant;
- e is the base of the natural logarithm;
- T is the total time of drug release;
- K is a constant incorporating factors such as the structural and geometric characteristics of the drug delivery system;
- n is the release exponent characterizing the release mechanism;
- M is the total amount of drug to be released;
- λ is the scale parameter;
- k is the shape parameter;
- $M_{t}$ and $M_{\infty }$ denote the cumulative amounts of drug released at time t and infinity, respectively;
- $k_{2}$ is the diffusion rate constant.

## Figures and Tables

**Table 1:** Correlation coefficients (r^2^) for the different formulations according to various models.

Model	r^2^	SD	*K* (µg/h)	SD	Formulation
Zero order	0.4864	0.0270	0.6276	0.0000	Carbopol
	0.6839	0.1963	1.0393	0.0000	Pluronic
	0.9642	0.0027	1.0103	0.0000	O/W
First order	0.8875	0.0808	0.0910	0.0111	Carbopol
	0.5868	0.1684	0.1147	0.0313	Pluronic
	0.9320	0.1018	0.0361	0.0007	O/W
Higuchi	0.7323	0.0302	1.6448	0.0517	Carbopol
	0.8869	0.0795	4.1211	1.5800	Pluronic
	0.8362	0.0573	9.5701	0.6970	O/W
Hixson–Crowell	0.9193	0.0610	0.0001	0.0000	Carbopol
	0.6956	0.2104	0.0003	−0.0002	Pluronic
	0.9639	0.0023	0.0003	0.0000	O/W
Korsmeyer–Peppas	0.7347	0.2225	1.5621	0.7230	Carbopol
	0.8203	0.1325	2.5891	1.1037	Pluronic
	0.9087	0.0384	4.5412	0.3306	O/W
Weibull	0.9615	0.0081	0.1317	0.0182	Carbopol
	0.9383	0.0285	0.4267	0.0508	Pluronic
	0.6774	0.0297	0.5144	0.0398	O/W
Baker–Lonsdale	0.7231	0.0240	0.0000	0.0000	Carbopol
	0.7694	0.1771	0.0000	0.0000	Pluronic
	0.9547	0.0250	0.0000	0.0000	O/W

**Table 2:** Composition of the EEt topical gel.

Ingredients	Carbopol	Pluronic 22%	Emulsion O/W
EEt	0.50 g	0.67 g	1.23 g
Carbopol^®^ 940	0.15 g	---	---
Triethanolamine	Enough to reach pH = 6.5 and 8.3	---	0.6 g
Pluronic F-127		6.6 g	---
Stearic acid	---	---	12 g
Glycerin	---	---	6.75 g
Methylparaben	---	---	0.05 g
Purified water	30 g	30 g	50 g

## Data Availability

Data are contained within the article.
